# CD38 Is Expressed on Inflammatory Cells of the Intestine and Promotes Intestinal Inflammation

**DOI:** 10.1371/journal.pone.0126007

**Published:** 2015-05-04

**Authors:** Michael Schneider, Valéa Schumacher, Timo Lischke, Karsten Lücke, Catherine Meyer-Schwesinger, Joachim Velden, Friedrich Koch-Nolte, Hans-Willi Mittrücker

**Affiliations:** 1 Institute of Immunology, University Medical Center Hamburg-Eppendorf, Hamburg, Germany; 2 Department of Internal Medicine, Nephrology, University Medical Center Hamburg-Eppendorf, Hamburg, Germany; 3 Institute for Pathology, University Medical Center Hamburg-Eppendorf, Hamburg, Germany; Charité-Universitätsmedizin Berlin, GERMANY

## Abstract

The enzyme CD38 is expressed on a variety of hematopoietic and non-hematopoietic cells and is involved in diverse processes such as generation of calcium-mobilizing metabolites, cell activation, and chemotaxis. Here, we show that under homeostatic conditions CD38 is highly expressed on immune cells of the colon mucosa of C57BL/6 mice. Myeloid cells recruited to this tissue upon inflammation also express enhanced levels of CD38. To determine the role of CD38 in intestinal inflammation, we applied the dextran sulfate sodium (DSS) colitis model. Whereas wild-type mice developed severe colitis, CD38^-/-^ mice had only mild disease following DSS-treatment. Histologic examination of the colon mucosa revealed pronounced inflammatory damage with dense infiltrates containing numerous granulocytes and macrophages in wild-type animals, while these findings were significantly attenuated in CD38^-/-^ mice. Despite attenuated histological findings, the mRNA expression of inflammatory cytokines and chemokines was only marginally lower in the colons of CD38^-/-^ mice as compared to wild-type mice. In conclusion, our results identify a function for CD38 in the control of inflammatory processes in the colon.

## Introduction

The nicotinamide adenine dinucleotide (NAD^+^) glycohydrolase CD38 is expressed on hematopoietic and non-hematopoietic cells. In the mouse, CD38^+^ hematopoietic cells include B cells, subsets of T cell, monocytes and macrophages. CD38 expression on these cells is modulated following activation and differentiation [1, 2]. CD38 is a type II transmembrane protein located on the cell surface or in intracellular vacuoles, with the enzymatic domain on the outside of the cell [1, 2]. There is also evidence for an inverse orientation placing the enzymatic activity into the cytosol [3]. CD38 catalyzes the formation of adenosine diphosphate ribose (ADPR) and nicotinamide from NAD^+^. CD38 has also ADPR cyclase as well as cyclic ADPR (cADPR) hydrolase activity resulting in the cADPR as a minor product. Under acidic conditions, CD38 can additionally generate nicotinic acid adenine dinucleotide phosphate (NAADP^+^) from NADP^+^ [4, 5, 6, 7]. ADPR, cADPR and NAADP^+^ are Ca^2+^ mobilizing second messengers. cADPR acts on ryanodine receptors and induces Ca^2+^ release from intracellular stores, ADPR activates the TRPM2 ion channel and induces influx of extracellular Ca^2+^, and NAADP^+^ targets acidic organelles like lysosomes [6, 7]. Via generation of these adenosine nucleotide second messengers, CD38 can modulate Ca^2+^ dependent activation and differentiation processes.

In the mouse, CD38 has been described as an activating co-receptor for B cells and modulates differentiation processes of these cells [1, 2]. On mouse neutrophils and dendritic cells, CD38 cooperates with several chemotactic receptors such as CCR2, CCR7, CXCR4 or N-formyl peptide receptors. CD38-mediated cADPR formation causes an increase in cytosolic Ca^2+^, which synergizes with signals from the chemotactic receptors in the induction of cell migration [8, 9, 10]. As a consequence, CD38-deficient neutrophils are less capable of accumulating at sites of bacterial infection [8, 11, 12], and CD38-deficient DCs fail to prime Th cells resulting in impaired T cell dependent antibody responses in mice [9]. CD38 is also the main hydrolase of extracellular NAD^+^ [1]. NAD^+^ released by stressed or damaged cells is a potential danger signal for immune cells [13, 14]. In the mouse, NAD^+^ is the substrate for ADP-ribosyl transferase 2 (ARTC2). ARTC2-mediated ADP-ribosylation of surface proteins on T cells causes either functional impairment of these proteins or in the case of the ion channel P2X7, constitutive activation with apoptosis as a main consequence. By reducing the concentration of extracellular NAD^+^, CD38 can restrict these processes [14, 15, 16].

In mouse infection models, absence of CD38 is associated with reduced innate anti-pathogen response, resulting in impaired control of bacteria and protozoa, but also with diminished immunopathology [8, 12, 17, 18, 19]. In several mouse models for autoimmunity and immunopathology, CD38^-/-^ mice demonstrate an ameliorated course of disease. CD38^-/-^ mice develop only mild joint inflammation in a collagen induced arthritis model [20], and show smaller lesion size after local ischemia and reperfusion in the brain [21]. In both models, CD38^-/-^ mice display reduced concentrations of pro-inflammatory cytokines and delayed cell recruitment to damaged tissues. CD38 is also necessary for manifestation of allergen-induced airway hyper-responsiveness in mice, and expression on both hematopoietic and non-hematopoietic cells is required for the development of this reaction [22]. In contrast, non-obese diabetic (NOD) mice deficient in CD38 show accelerated development of type-1 diabetes, which is most likely due to ARTC2-mediated deletion of protective NKT cells [23, 24]. Overall, these results indicate a regulatory role for CD38 in both innate and acquired immune responses.

In a recent study, we detected high expression levels of CD38 on immune cells of the intestinal mucosa [15]. We therefore hypothesized that CD38 might influence inflammatory processes in the intestine. To test this hypothesis, we treated CD38^-/-^ mice with DSS and analyzed the inflammatory response in the colon mucosa.

## Material and Methods

### Mice

CD38^-/-^ [25] mice were backcrossed for 12 generations to the C57BL/6 background. All mice were bred under specific pathogen-free conditions in the animal facility of the University Medical Center Hamburg-Eppendorf. Experiments were performed according to state guidelines and approved by the local ethics committee (Registration number: 21/09).

### DSS-induced intestinal inflammation

Mice received 3% DSS (dextran sulfate sodium) dissolved in the drinking water. DSS with a molecular weight of 36–50 kDa (MP Biomedicals, Eschwege, Germany) was used. After 5 days, DSS water was replaced by regular water. Weight of mice was determined daily. Unless stated otherwise, mice were killed 7 days after the start of DSS treatment and tissues of mice were analyzed. At this time point, mice were assessed for clinical signs of disease using a semi-quantitative score [26, 27]: normal stool pellets, no blood 0; soft stool pellets, no blood 1; soft stool pellets, stool with blood 2; no stool pellet formation, stool with blood 3; no stool pellet formation, massive rectal bleeding 4. The length of the colon from the colo-cecal junction to the anal verge was measured and divided by whole-animal body weight as a rough macroscopic estimate of inflammation.

### Cell isolation

Cells from mouse spleens were obtained by mashing the disintegrated organs through cell strainers into PBS. This was followed by erythrocyte lysis with ACK lysing buffer (155 mM NH_4_Cl, 10 mM KHCO_3_, 100 **μ**M EDTA, pH ~7.2). Intestinal leukocytes from the epithelium of the large intestine (colon and cecum) were isolated as follows: Large intestines were cut open longitudinally and washed in PBS. Tissues were stirred for 30 min at 37°C in RPMI 1640 medium supplemented with β2-mercaptoethanol, gentamycin, glutamine and FCS. The supernatant was collected and passed through a cell strainer. Leukocytes were purified by running a 40%/70% percoll gradient (EasyColl, Biochrom, Berlin, Germany). To obtain lamina propria lymphocytes, the remainders of the large intestines were cut into small pieces and digested with 0.25 mg/ml Collagenase D (Roche, Mannheim, Germany), 0.25 mg/ml Collagenase VIII from *Clostridium histolyticum* (Sigma Aldrich, St. Louis, MO) and 10 U/ml DNaseI (Sigma Aldrich) in total 5 ml RPMI 1640 for 30 min at 37°C while shaking [15].

### Flow cytometric analysis of cells

For surface staining, cells were first incubated with 10 μg/ml 2.4G2 (anti-FcγRII/III; BioXCell, West Lebanon, NH) and 1:100 normal rat serum to minimize unspecific antibody binding. Staining was performed on ice with monoclonal antibodies (mAb) conjugated to FITC, PE, PerCP, PE-Cy7, APC, APC-Cy7, or V450 according to standard methods: anti-CD4 mAb (clones RM4-5), anti CD8α mAb (53–6.7), anti CD8β mAb (H35-17.2), anti-CD11b mAb (M1/70), anti CD19 mAb (1D3), anti-CD38 mAb (90), anti-CD45 mAb (30-F11), anti-CD157 mAb (BP-3), anti-Ly6GC/Gr-1 mAb (RB6-8C5) and anti-Ly6C mAb (HK1.4). Antibodies were purchased from eBioscience (San Diego, CA), BioLegend (San Diego, CA) or BD Biosciences (San Jose, CA). Cells were analyzed on the CantoII flow cytometer (BD Biosciences) after gating out doublets and DAPI^+^ dead cells. Data were analyzed with FACS Diva software (BD Biosciences) or FlowJo software (Treestar, Ashland, OR).

### RNA purification and quantitative PCR

Sections of spleen and colon were snap frozen in Trizol reagent (Invitrogen, Karlsruhe, Germany) and stored at -80°C. After the addition of tungsten beads, tissues were homogenized with a TissueLyser device (Qiagen, Venlo, Germany). RNA was purified from homogenates using the protocol provided for the Trizol reagent and stored in DEPC water in the presence of RNAse inhibitors (RNAse out, Invitrogen, Karlsruhe, Germany). RNA was transcribed into cDNA with M-MLV reverse transcriptase and random hexamer oligonucleotides (Invitrogen, Karlsruhe, Germany). Quantitative PCR was conducted using SYBR green with primers as provided in Table 1. *IL17a* was determined with the Taqman method with a commercial primer set (Mm00439618_m1; Life Technologies, Carlsbad CA) Samples were measured in duplicates with a StepOnePlus thermocycler (Applied Biosystems, Foster City, CA) and analyzed with the software provided with the thermocycler. Values were normalized to values for 18S rRNA and are presented as ΔΔCT values in relation to the values of samples of untreated wild type animals.

**Table 1:** 

Primers for quantitative PCR
18S forward CAC GGC CGG TAC AGT GAA AC
18S reverse AGA GGA GCG AGC GAC CAA A
*Cxcl1* forward GCA CCC AAA CCG AAG TCA TAG
*Cxcl1* reverse CAA GGG AGC TTC AGG GTC AA
*Cxcl2* forward CAC TGC GCC CAG ACA GAA
*Cxcl2* reverse CAG GGT CAA GGC AAA CTT TTT G
*Ccl2* forward GGC TCA GCC AGA TGC AGT TAA
*Ccl2* reverse CCT ACT CAT TGG GAT CAT CTT GCT
*Tnfalpha* forward AAA TGG CCT CCC TCT CAT CAG T
*Tnfalpha* reverse GCT TGT CAC TCG AAT TTT GAG AAG
*Il6* forward TGG GAA ATC GTG GAA ATG AGA
*Il6* reverse AAG TGC ATC ATC GTT GTT CAT ACA
*Il10* forward GCT GCG GAC TGC CTT CAG
*Il10* reverse AGG AGT CGG TTA GCA GTA TGT TGT C
*IL23p19* forward ATC CAG TGT GAA GAT GGT TGT GA
*IL23p19* reverse CGG ATC CTT TGC AAG CAG AA

### Histology and immunohistology

Samples from the middle segment of the colon were fixed in 4% w/v paraformaldehyde for 24 h, washed with PBS and embedded in paraffin. Sections (3 μm) were rehydrated by running through xylen, and ethanol series of decreasing concentrations and finally water. Sections were stained with hematoxylin and eosin. Sections were scored in a blinded fashion for infiltration and alterations of epithelium and crypt morphology [26]. Grade of infiltration: rare infiltration by inflammatory cells 0, crypt infiltration 1, infiltration reaching the muscularis mucosa 2, thickening of the mucosa 3, infiltration extending to the submucosa 4. Grade of alteration of epithelium and crypts: normal morphology 0, loss of goblet cells 1, loss of goblet cells in large areas 2, loss of crypts 3, loss of crypts in large areas 4. Values from both grading schemes were added up to a total histological score.

For immunohistology, sections were rehydrated. Ag retrieval was performed by incubation with proteinase XXIV (5 mg/ml; Sigma-Aldrich, Steinheim, Germany, and St. Louis, MO) for anti-Ly6G mAb staining and for 15 min at 37°C or with trypsin (1 mg/ml; Sigma-Aldrich, Steinheim, Germany, and St. Louis, MO) for 10 min at 37°C for anti-F4/80 mAb staining. After blocking for 30 min in 5% horse serum (Vector), the tissue was incubated with rat anti-Ly6G mAb (1:25; Hycult, Uden, The Netherlands) or rat anti-F4/80 mAb (1:300, BM8; eBioscience, San Diego, CA) in 5% horse serum for 1 h at room temperature or overnight at 4°C. Staining was visualized using biotinylated affinity-purified secondary Abs (Jackson ImmunoResearch Laboratories) diluted 1:200 in 5% horse serum for 30 min, followed by the ABC kit (Vector Laboratories, Burlingame, CA), according to the manufacturer’s instructions. Sections were counterstained with hematoxylin. Samples were evaluated under an Axioskop (Zeiss, Jena, Germany) and photographed with an Axiocam HRc (Zeiss) [28].

### Statistical analysis

All statistical analysis was performed with Prism software (GraphPad Software Inc., La Jolla, CA). In the graphs each mouse is presented by a single symbol. A line indicates the median value for all animals within one experimental group. Differences between groups of mice were analyzed by Mann-Whitney U test. A p-value of <0.05 was considered significant (*: p<0.05; **: p<0.01; ***: p<0.001; ns: not significant).

## Results

### CD38 expression on leukocytes of the colon mucosa

**Fig 1:**
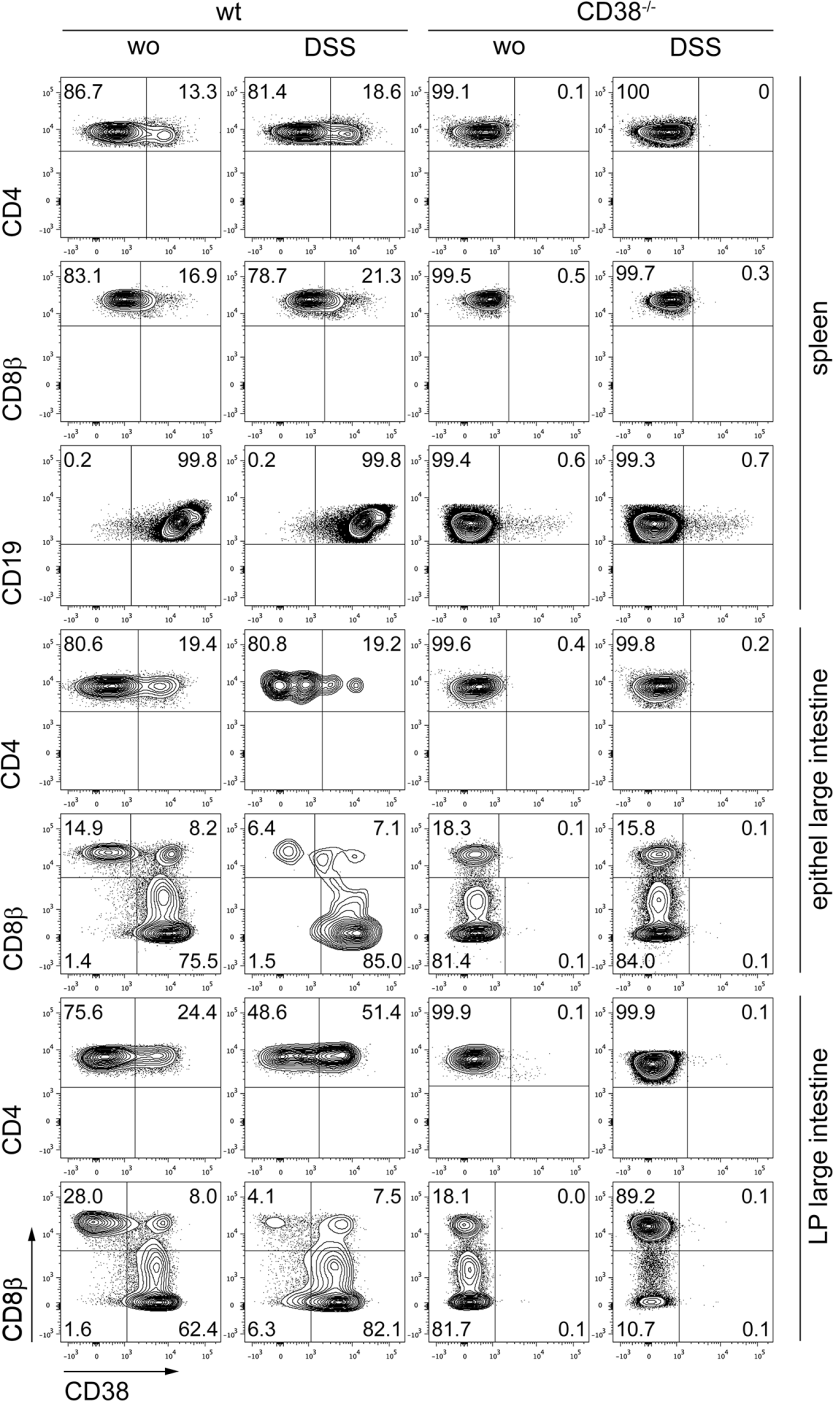
Expression of CD38 on T cells. Wild-type and CD38^-/-^ mice received 3% DSS in the drinking water or were left untreated (wo). After 5 days, DSS water was replaced by normal tap water. On day 7, cells were isolated from spleen as well as large intestine epithelium and lamina propria (LP) and analyzed by flow cytometry. Blots show CD38 expression on viable CD45^+^ CD4^+^ and CD8α^+^ T cells. For the spleen, only CD8αβ^+^ T cells are shown. Viable CD45^+^CD19^+^ B cells are only presented for the spleen. For the large intestine, CD8α^+^ T cells are further separated into conventional CD8αβ^+^ and unconventional CD8αα^+^ (CD8α^+^β^-^) T cells. Dot blots give representative results for cells pooled from 5 mice per group.

**Fig 2:**
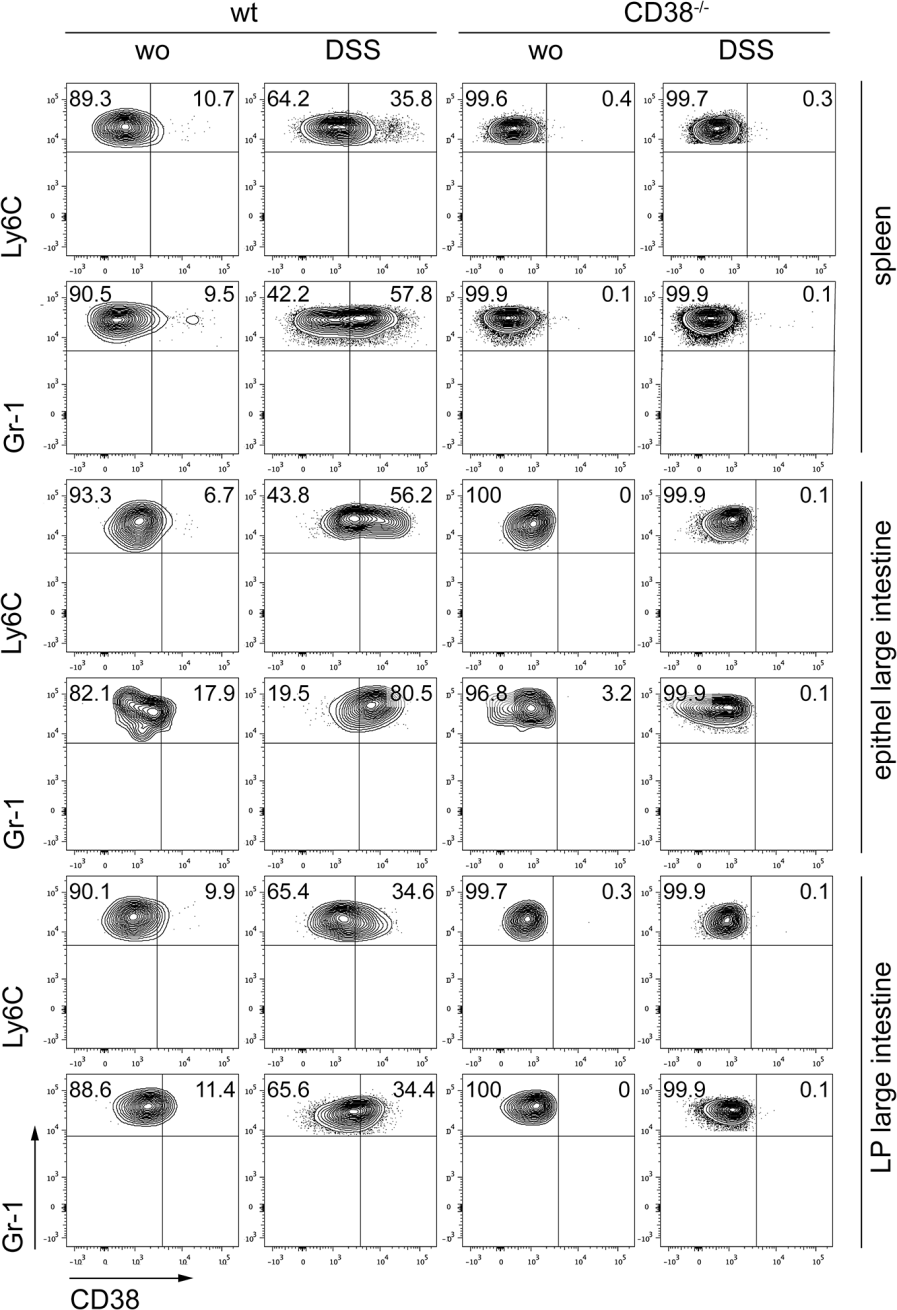
Expression of CD38 on granulocytes and inflammatory monocytes. Wild-type and CD38^-/-^ mice received 3% DSS in the drinking water or were left untreated (wo). After 5 days, DSS water was replaced by normal tap water. On day 7, cells were isolated from spleen as well as large intestine epithelium and lamina propria (LP) and analyzed by flow cytometry. Blots show CD38 expression on viable CD45^+^ granulocytes (CD11b^+^Gr-1^high^ cells) and inflammatory monocytes (CD11b^+^Ly6C^high^ cells). Dot blots give representative results for cells pooled from 5 mice per group.

In a first set of experiments, we determined the cell surface levels of CD38 on T cells and on myeloid cells rapidly recruited to inflamed tissues, i.e. granulocytes and inflammatory monocytes, 7 days after initiation of DSS treatment. In comparison to B cells with high CD38 surface expression, most CD4^+^ and CD8^+^ T cells from the spleen of naive mice expressed only low CD38 levels or were CD38-negative (Fig 1). Spleen granulocytes (CD11b^+^ Gr-1^hi^ Ly6C^lo^) and inflammatory monocytes (CD11b^+^ Gr-1^lo^ Ly6C^hi^) also showed low cell surface levels of CD38 (Fig 2). Treatment of mice with DSS only marginally changed the CD38 expression profile of spleen T cells, but caused up-regulation of CD38 on inflammatory monocytes and granulocytes. Only a subpopulation of CD4^+^ T cells (~20%) from large intestine epithelium and lamina propria of naive wild-type mice was CD38^+^. This subpopulation was enlarged in the lamina propria after DSS treatment (Fig 1). In large intestine epithelium and lamina propria, conventional CD8^+^ T cells, as defined by co-expression of CD8α and CD8β, were divided in CD38-negative cells and in cells with high expression of the molecule (20–30% of CD8αβ^+^ T cells). After DSS treatment, more than half of the conventional CD8αβ^+^ T cells presented with a CD38^+^ phenotype. In contrast, already in naive mice virtually all unconventional CD8αα^+^ T cells (CD8α^+^β^-^) expressed high levels of CD38 (Fig 1). In large intestine epithelium and lamina propria of naive mice, granulocytes and inflammatory monocytes presented with a CD38 expression profile similar to that observed in the spleen (Fig 2). DSS treatment caused up-regulation of CD38 in both cell subsets, which was particularly strong in the epithelium where more than half of the inflammatory monocytes and 80% of the granulocytes became CD38^+^ (Fig 2). In conclusion, our results reveal expression of CD38 on subsets of resident large intestine T cells and on granulocytes and inflammatory monocytes recruited to the inflamed intestine.

CD157 is a homologue of CD38 and is also able to metabolize NAD^+^ to cADPR and ADPR [2, 29]. T cells, inflammatory monocytes and granulocytes were therefore analyzed for the expression of CD157. T cells from spleen and large intestine lamina propria of naive and DSS treated wild-type and CD38^-/-^ mice were CD157-negative (S1 Fig). In spleen and large intestine, inflammatory monocytes expressed low and granulocytes high levels of CD157. On both cell subsets, CD157 was up-regulated following DSS treatment (S2 Fig). Absence of CD38 had only marginal effects on CD157 expression levels.

### CD38-deficiency protects mice from DSS-induced intestinal inflammation

**Fig 3:**
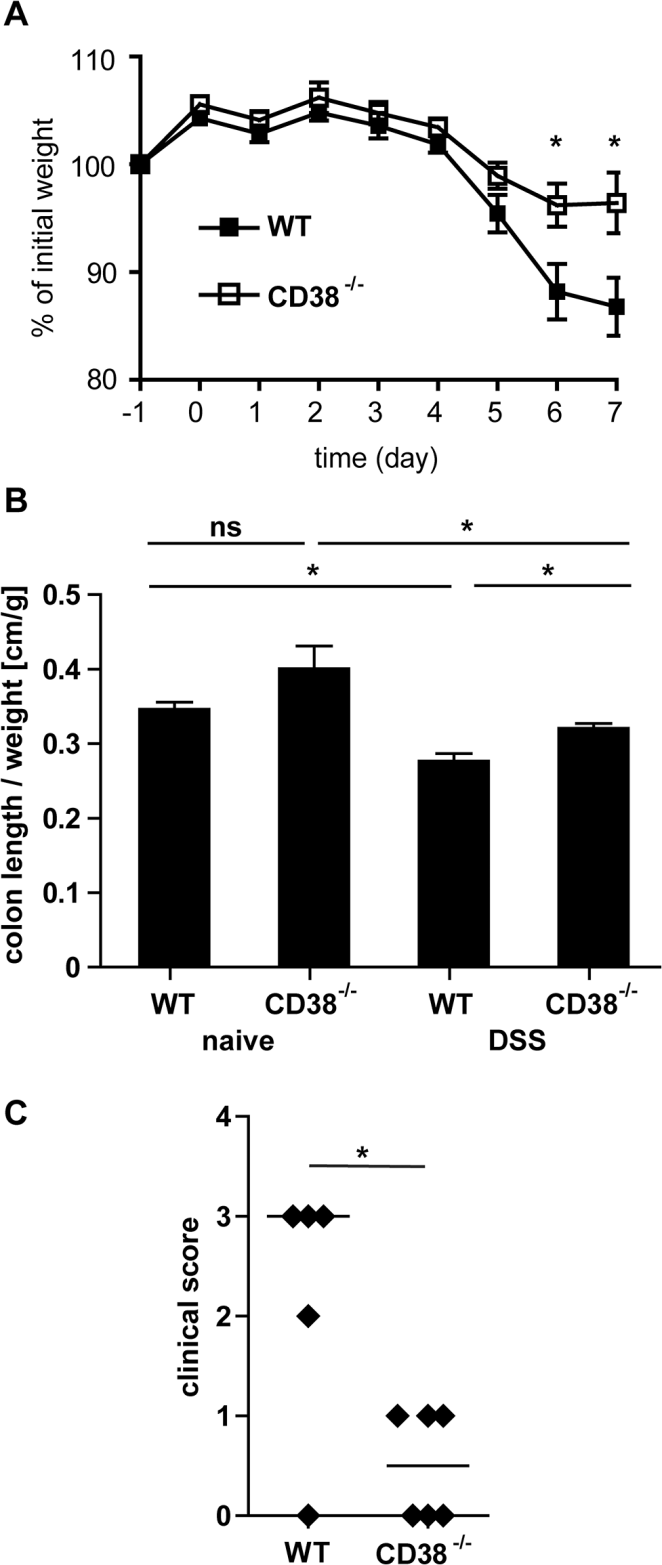
CD38^-/-^ mice develop only mild colitis in response to DSS-treatment. Wild-type and CD38^-/-^ mice were treated with DSS as in Fig 1. On day 7, mice were killed and colons were analyzed. (A) Weight changes of wild-type and CD38^-/-^ mice after DSS treatment. Weight was determined daily and is given as % of weight one day before the beginning of DSS treatment. Results represent the mean ± SEM of 6 individually analyzed mice per group. (B) Relative colon length of untreated and DSS-treated wild-type and CD38^-/-^ mice. Colon length was normalized to the whole-animal body weight, which was determined before DSS treatment. Bars give the mean ± SEM of 5 or 6 individually analyzed mice per group. (C) Clinical score of DSS-treated wild-type and CD38^-/-^ mice at day 7. Parameters of scoring are given in the method section. Values for individually analyzed mice and the median are shown. * p<0.05; ns not significant (p>0.05)

DSS treatment caused profound weight loss in wild-type mice (Fig 3A) accompanied by clinical signs of disease and by a shortening of the colon due to inflammation (Fig 3B and 3C). DSS-treated CD38^-/-^ mice showed significantly less weight loss than wild-type mice (Fig 3A), less shortening of the colon and milder clinical disease scores (Fig 3B and 3C).

**Fig 4:**
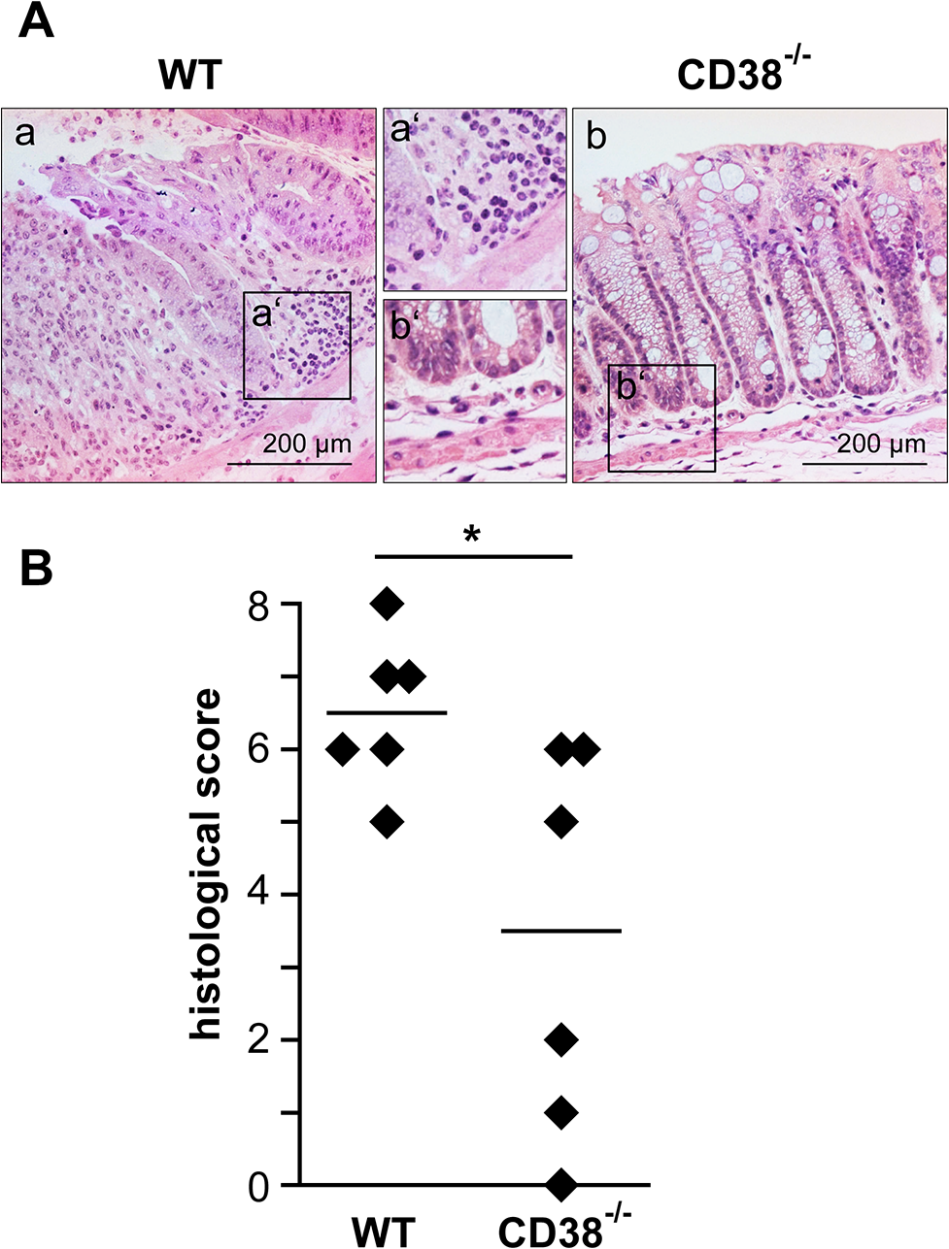
Morphological changes in the colons of wild-type and CD38^-/-^ mice after DSS treatment. Wild-type and CD38^-/-^ mice were treated with DSS as in Fig 1. On day 7, mice were killed and colon sections were H&E stained. (A) Representative H&E stained sections of colons from DSS-treated wild-type (a, a’) and CD38^-/-^ mice (b, b’). Original magnification: 200×. (B). Colon sections were evaluated for tissue damage (score 0–4) and for cellular infiltration (score 0–4), and both scores were added up to a histological score. Parameters of scoring are given in the method section. The figure gives results for individual mice and the median of 6 mice per group. * p<0.05.

**Fig 5:**
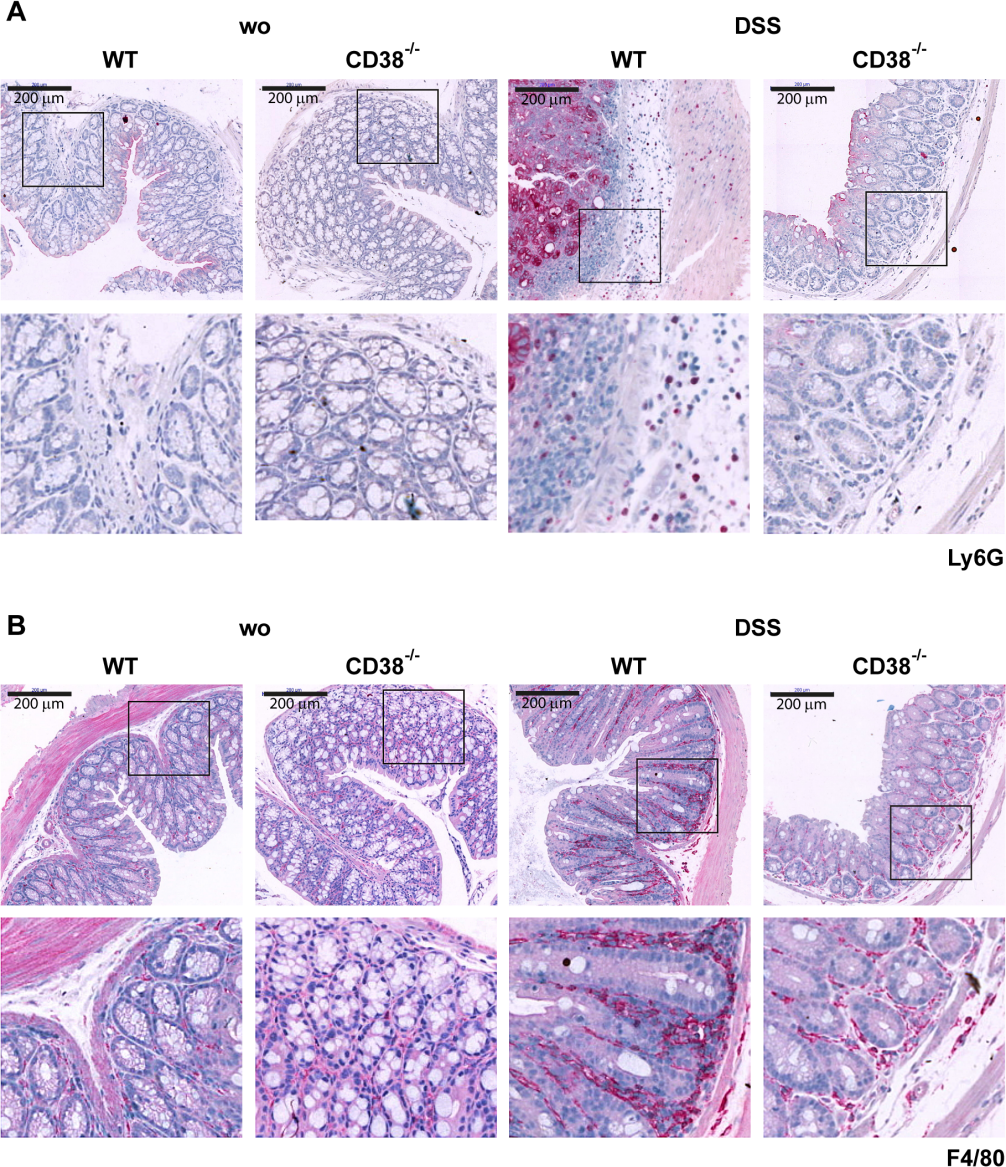
Accumulation of granulocytes and macrophages in colons of DSS-treated mice. Wild-type and CD38^-/-^ mice were treated with DSS as in Fig 1. On day 7, mice were killed and colon sections were immunohistochemically stained (red) with anti-Ly6G mAb (A) or anti-F4/80 mAb (B). Strong Ly6G signals close to the lumen are due to unspecific staining of the epithelial glycocalix. Representative figures are shown. Original magnification: 100× and 400×.

Histological sections of the colon from DSS-treated wild-type and CD38^-/-^ mice were analyzed for cellular infiltration and alterations in the morphology of the epithelium, crypts and submucosa (Fig 4A). Scoring of these parameters revealed severe inflammation in wild-type mice (Fig 4B). In contrast, CD38^-/-^ mice presented with significantly reduced colonic inflammation and damage scores. Immunohistology further indicated that infiltration of the colonic mucosa by both granulocytes and macrophages was less pronounced in CD38^-/-^ mice as compared to wild-type mice (Fig 5). Taken together, these results indicate that CD38 mice are substantially less susceptible to DSS-induced colitis.

### Production of inflammatory cytokines in the colon of CD38^-/-^ mice

**Fig 6:**
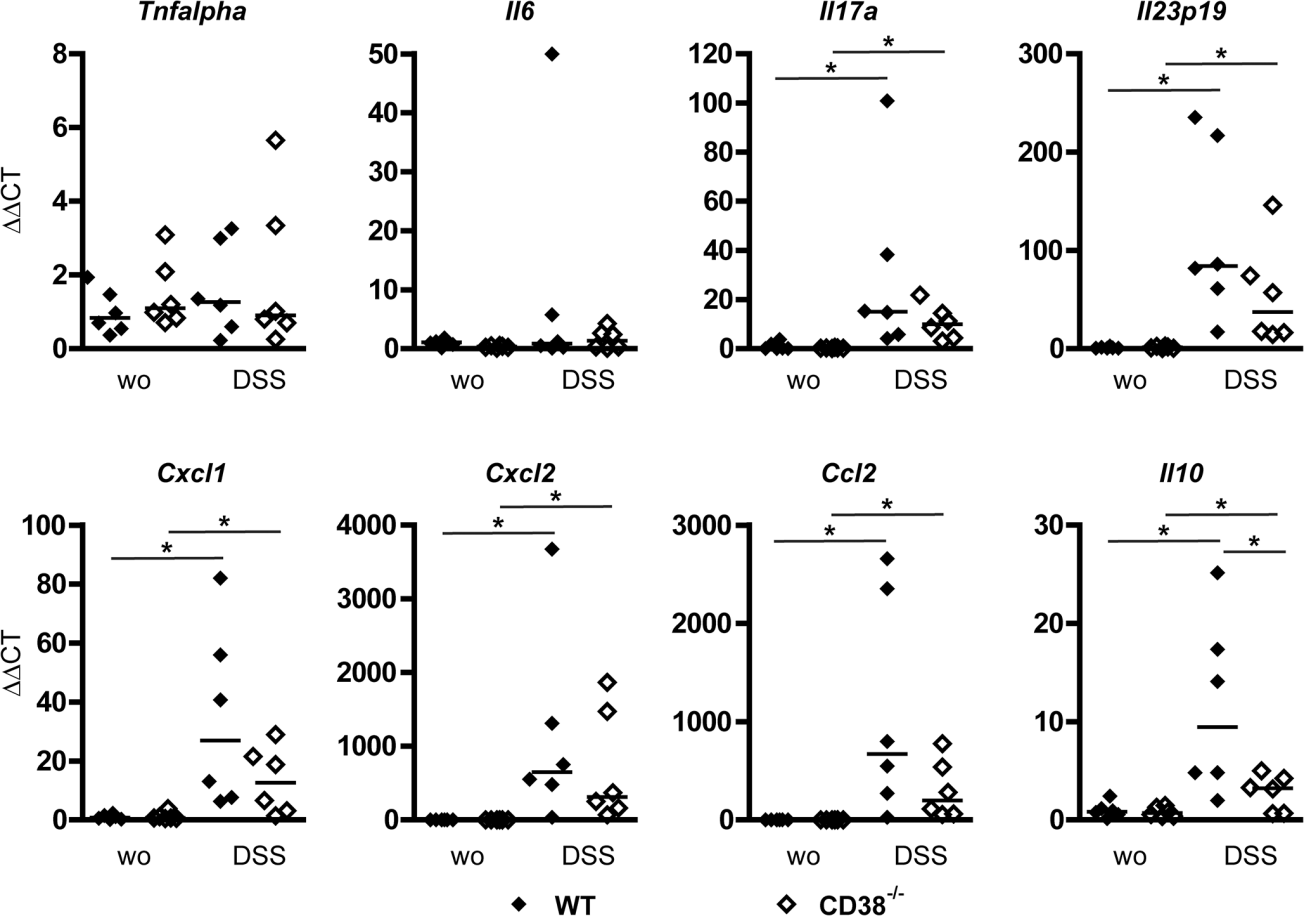
Production of inflammatory cytokines in colons of DSS-treated wild-type and CD38^-/-^ mice. Wild-type and CD38^-/-^ mice were treated with DSS as in Fig 1. On day 7, mice were killed and colon tissue extracts were analyzed for the mRNA expression levels of cytokines and chemokines. Values were calibrated to the corresponding 18S rRNA values and are presented as ΔΔCT values normalized to the mean values of untreated wild-type mice. Graphs present values of 6 individual mice per group and the median. * p<0.05.

To assess the impact of CD38 on intestinal production of inflammatory cytokines and chemokines, wild-type and CD38^-/-^ mice were treated with DSS and mRNA expression levels were determined in colon tissue extracts (Fig 6). For *Tnfalpha*, we observed only marginal changes in the expression level in the colons of untreated and DSS-treated mice, and wild-type and CD38^-/-^ mice showed similar expression of the cytokine. This somewhat surprising result could be explained by the relatively high basal expression levels of *Tnfalpha* in the colon of untreated mice (as compared to the 18S rRNA levels; not shown). In the spleen, where basal *Tnfalpha* expression levels were lower, DSS treatment caused significant up-regulation both in wild-type and CD38^-/-^ mice (data not shown). In the colon, *Il6* transcript levels also revealed only minimal differences in most DSS-treated vs. untreated mice of either genotype. *Il17a* and *Il23p19* transcript levels were significantly upregulated in colons of both genotypes following DSS-treatment, and although the expression of both these cytokines appeared slightly lower in CD38^-/-^ than in wild-type mice, the difference was not significant. DSS treatment caused strong up-regulation of the neutrophil chemokines *Cxcl1* and *Cxcl2*, as well as of the chemokine *Ccl2* (*Mcp1*), which attracts monocytes. Again, there was a tendency towards lower expression levels of these chemokines in DSS-treated CD38^-/-^ mice vs. wild-type mice. Finally, mRNA for the anti-inflammatory cytokine *Il10* was significantly upregulated by DSS-treatment in the colons of both genotypes, however, expression levels were significantly lower in CD38^-/-^ mice. In summary, absence of CD38 did not prevent the induction of inflammatory cytokines and chemokines, although there was a common trend towards lower expression of both inflammatory and anti-inflammatory cytokines in CD38^-/-^ mice as compared to wild-type mice.

## Discussion

In the epithelium and lamina propria of the large intestine, subpopulations of conventional CD4^+^ and CD8αβ^+^ T cells showed relatively high surface expression of CD38. These subpopulations were enlarged following DSS treatment. CD38 expression on conventional intestinal T cells could be a result of activation or of preferential recruitment of CD38^+^ cells following DSS treatment. CD38 expression could also be part of the intestinal phenotype imprinted on T cells during priming in the gut mucosa [30]. Production of retinoic acid by intestinal dendritic cells is considered central in this process [31, 32] and CD38 is induced in different cell types, including T cells, by retinoic acid ([33, 34, 35] and own unpublished observation). Imprinting of CD38 could also explain the relatively high CD38 expression level on conventional intestinal T cells, already under steady state conditions. Unconventional CD8αα^+^ T cells were homogenously CD38^high^. The cause of the high CD38 expression level on these T cells is unclear. CD38 expression could be part of their differentiation program or due to signals from the intestinal environment. Induction of CD38 by retinoic acid has also been described for human T cells, and T cells from the human lamina propria express high levels of CD38 [33, 36]. In peripheral blood, CD38 expression on pre-activated CD62L^low^ CD4^+^ T cells correlates with CCR9 and particularly with β7-integrin expression, and gliadin-specific CD4^+^ T cells from patients with celiac disease are homogenously CD38^+^ [33]. Therefore, CD38 expression has even been proposed to be a marker for CD4^+^ T cells primed in the intestinal mucosa [33]. High CD38 expression levels on T cells from intestinal mucosa indicate a particular function for CD38 in this tissue. Via its enzymatic activity, CD38 could reduce extracellular NAD^+^ and protect T cells from detrimental effects of the nucleotide (see below). CD38 could also directly influence responses of intestinal T cells. Interestingly, CD38 stimulation causes activation of human lamina propria T cells that are refractory to TCR stimulation [36]. On the other hand, following CD38 stimulation, T cells from the human lamina propria fail to show an increase of cytoplasmic Ca^2+^ and display an altered cytokine production pattern when compared to T cells from human peripheral blood [36]. Thus, CD38 might participate in a distinct activation program of T cells in the intestinal mucosa. In contrast to the situation in the human, very little is known about the function of CD38 on mouse T cells and it remains to be determined to which degree results for human CD38 can be transferred to the mouse system.

Following DSS treatment, meutrophils and inflammatory monocytes recruited to the intestinal mucosa showed relatively high levels of cell surface CD38. We and others have previously shown that granulocytes and monocytes upregulate CD38 expression at sites of inflammation [8, 12]. Thus high expression levels are most likely a consequence of activation, however, we cannot exclude that the intestinal environment also causes enhanced CD38 expression on these cells.

CD157 was not detected on T cells. In accordance with published results, inflammatory monocytes expressed low levels and granulocytes high levels of CD157 [2, 37], and both cells further increased expression following DSS treatment. CD157 can metabolize NAD^+^ to cADPR and ADPR and thereby could cooperate with CD38 in local nucleotide metabolism. Indeed, it has been reported that paneth cells of the intestinal mucosa can upregulate CD157 resulting in local cADPR production [38]. However, compared to CD38, CD157 has only very low enzymatic activity and in tissues of CD38^-/-^ mice, NAD^+^ glycohydrolase activity was undetectable [25, 39]. Furthermore, CD157 is a GPI-linked surface protein precluding direct transmembrane signaling [2, 37]. Therefore, CD157 and CD38 most likely have distinct functions and CD157 probably cannot functionally compensate for the lack of CD38 in CD38^-/-^ mice.

Compared to wild-type mice, CD38^-/-^ mice showed reduced weight loss and only mild colon inflammation upon DSS treatment. These results imply that CD38 contributes to the development of DSS-induced colitis. This is consistent with observations in other models of inflammatory disorder where CD38^-/-^ mice also show only mild disease [20, 21, 22]. In these models, attenuated inflammation resulted from reduced production of cytokines and chemokines, as well as reduced migration of inflammatory cells [20, 21, 22]. We observed reduced accumulation of granulocytes and inflammatory monocytes in the colon of DSS-treated CD38^-/-^ mice vs. wild-type mice, concomitant with a trend towards lower transcript levels of CXCL1, CXCL2 and CCL2. Thus, defects in chemokine production and cell recruitment could be responsible for reduced colon inflammation. Interestingly, TRPM2^-/-^ mice also display only mild inflammation in the DSS colitis model [40]. Following DSS treatment, TRPM2^-/-^ mice respond with reduced production of CXCL2 and diminished neutrophil accumulation in the colon. In contrast, expression of monocyte-attracting chemokines and recruitment of monocytes is only marginally affected [40]. Since TRMP2 is regulated by the CD38 product ADPR, these results suggest that the CD38-ADPR-TRPM2 axis might play a central role in colon inflammation.

CD38 is an important hydrolase for extracellular NAD^+^ [2, 25] and particularly in the intestinal mucosa, CD38 might be necessary to counter excessive NAD^+^ concentrations. We have recently demonstrated that lymphocytes of the intestinal mucosa express high levels of ARTC2 and P2X7 and are therefore sensitive to ADP-ribosylation of cell surface proteins and to NAD^+^ induced cell death subsequent to activation of P2X7 [15, 41]. In DSS-treated CD38^-/-^ mice, extracellular NAD^+^ could reach concentrations sufficient to trigger these processes in intestinal T cells. The impact of ARTC2-mediated ADP-ribosylation on colon inflammation remains unclear. Inactivation and deletion of pro-inflammatory T cells could dampen mucosal inflammation. However, activated T cells rapidly shed ARTC2 from the surface and become insensitive to extracellular NAD^+^ [42]. On the other hand, regulatory T cells, which play a central role in mucosal integrity, are particularly sensitive to extracellular NAD^+^ [43]. Thus, ARTC2 might influence both pro- and anti-inflammatory processes in the presence of high extracellular NAD^+^ concentrations.

In summary, we demonstrate a fundamental role of CD38 in intestinal inflammation. The similar phenotype of CD38^-/-^ and TRPM2^-/-^ mice in the DSS colitis model further suggests that ADPR generation is central to this function of CD38. These results also indicate that targeting CD38 activity could offer a new therapeutic option for the treatment of inflammatory bowel diseases such as Crohn's disease or ulcerative colitis.

## Supporting Information

S1 Fig Expression of CD157 on T cells. Wild-type and CD38^-/-^ mice received 3% DSS in the drinking water or were left untreated (wo). After 5 days, DSS water was replaced by normal tap water. On day 7, cells were isolated from spleen and lamina propria (LP) of the large intestine and analyzed by flow cytometry. Blots show CD157 expression on viable CD45^+^ CD4^+^ and CD8α^+^ T cells from naive and DSS treated mice. For the spleen, only CD8αβ^+^ T cells are shown. For the large intestine, CD8α^+^ T cells are further separated into conventional CD8αβ^+^ and unconventional CD8αα^+^ (CD8α^+^β^-^) T cells. CD157 expression was correlated to an isotype control staining of spleen T cells. Dot blots give representative results for cells pooled from 5 mice per group.
(PDF)

S2 Fig Expression of CD157 on inflammatory monocytes and granulocytes. Wild-type and CD38^-/-^ mice received 3% DSS in the drinking water or were left untreated (wo). After 5 days, DSS water was replaced by normal tap water. On day 7, cells were isolated from spleen and lamina propria (LP) of the large intestine and analyzed by flow cytometry. Blots show CD157 expression on viable CD45^+^ granulocytes (CD11b^+^Gr-1^high^ cells) and inflammatory monocytes (CD11b^+^Ly6C^high^ cells) from naive and DSS treated mice. CD157 expression was correlated to an isotype control staining of spleen cells. Dot blots give representative results for cells pooled from 5 mice per group.
(PDF)
