# Supplementary figures and images for: CD38 Is Expressed on Inflammatory Cells of the Intestine and Promotes Intestinal Inflammation
Source: PLoS One. 2015 May 4;10(5):e0126007. doi: 10.1371/journal.pone.0126007 (PMC4418770; doi:10.1371/journal.pone.0126007)

# Supplementary Figure 1

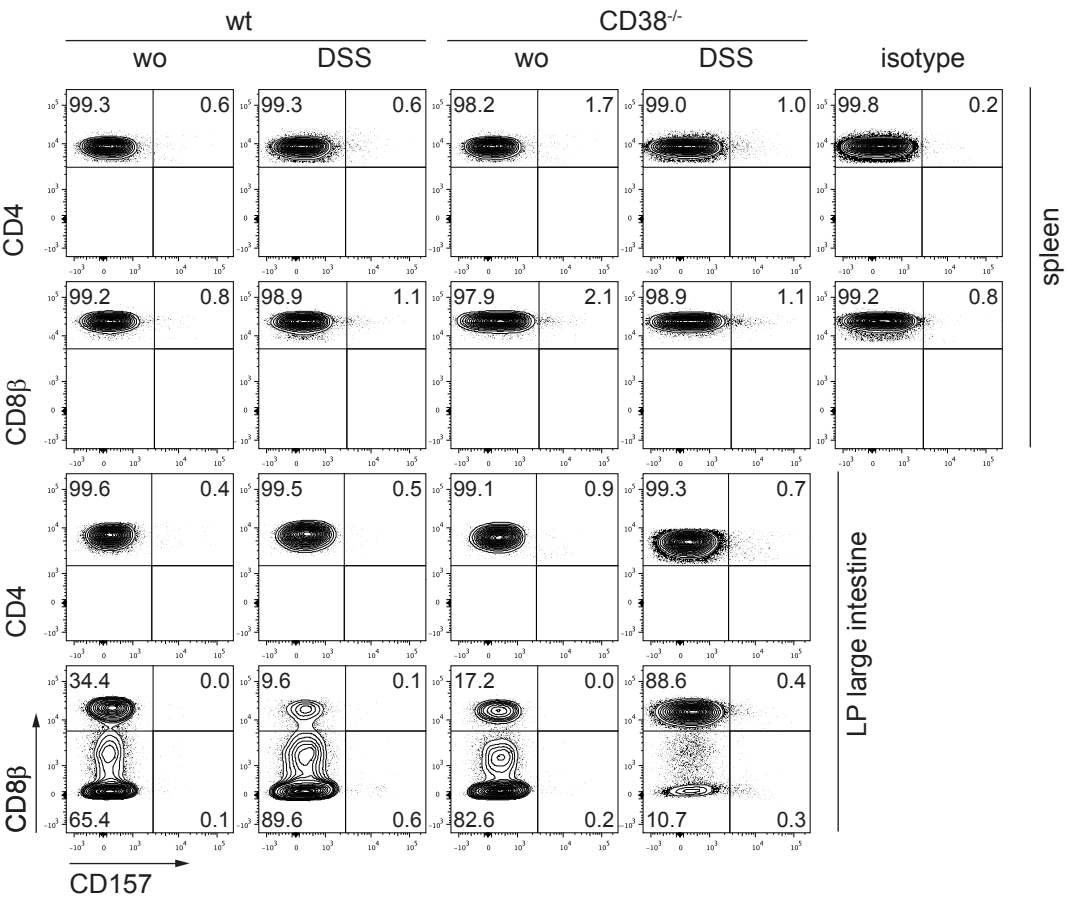

Supplement: S1 Fig — Wild-type and CD38-/- mice received 3% DSS in the drinking water or were left untreated (wo). After 5 days, DSS water was replaced by normal tap water. On day 7, cells were isolated from spleen and lamina propria (LP) of the large intestine and analyzed by flow cytometry. Blots show CD157 expression on viable CD45+ CD4+ and CD8α+ T cells from naive and DSS treated mice. For the spleen, only CD8αβ+ T cells are shown. For the large intestine, CD8α+ T cells are further separated into conventional CD8αβ+ and unconventional CD8αα+ (CD8α+β-) T cells. CD157 expression was correlated to an isotype control staining of spleen T cells. Dot blots give representative results for cells pooled from 5 mice per group. (PDF) [file pone.0126007.s001.pdf]

# Supplementary Figure 2

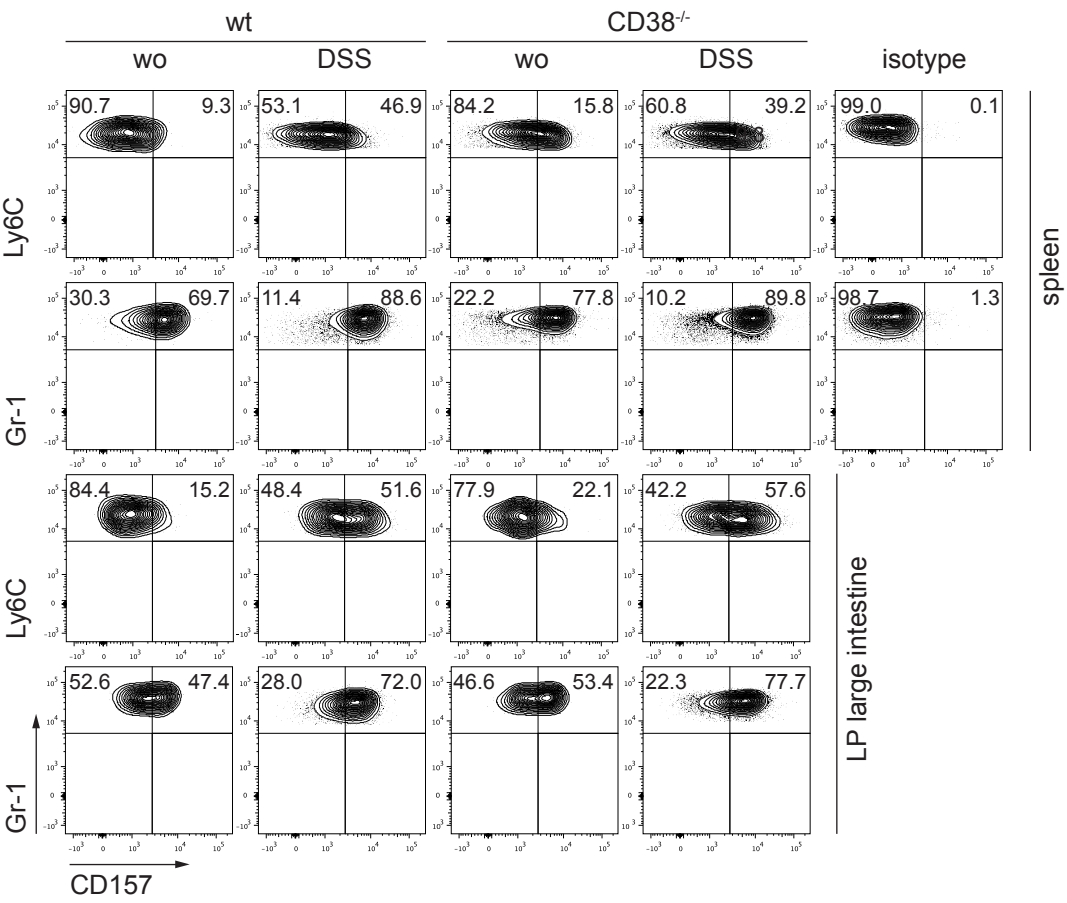

Supplement: S2 Fig — Wild-type and CD38-/- mice received 3% DSS in the drinking water or were left untreated (wo). After 5 days, DSS water was replaced by normal tap water. On day 7, cells were isolated from spleen and lamina propria (LP) of the large intestine and analyzed by flow cytometry. Blots show CD157 expression on viable CD45+ granulocytes (CD11b+Gr-1high cells) and inflammatory monocytes (CD11b+Ly6Chigh cells) from naive and DSS treated mice. CD157 expression was correlated to an isotype control staining of spleen cells. Dot blots give representative results for cells pooled from 5 mice per group. (PDF) [file pone.0126007.s002.pdf]
